# Predicting the Refractive Index of Tissue Models Using Light Scattering Spectroscopy

**DOI:** 10.1177/0003702820984482

**Published:** 2021-01-19

**Authors:** Michelle Bailey, Benjamin Gardner, Martina Alunni-Cardinali, Silvia Caponi, Daniele Fioretto, Nick Stone, Francesca Palombo

**Affiliations:** 1 School of Physics and Astronomy, University of Exeter, Exeter, UK; 2 Department of Physics and Geology, University of Perugia, Perugia, Italy; 3 CNR-IOM – Istituto Officina dei Materiali – Research Unit in Perugia, c/o Department of Physics and Geology, University of Perugia, Perugia, Italy

**Keywords:** Biopolymers, biomechanics, vibrational spectroscopy, Brillouin microscopy, Raman scattering

## Abstract

In this work, we report the application of Raman microspectroscopy for analysis of the refractive index of a range of tissue phantoms. Using both a custom-developed setup with visible laser source and a commercial microspectrometer with near infrared laser, we measured the Raman spectra of gelatin hydrogels at various concentrations. By building a calibration curve from measured refractometry data and Raman scattering intensity for different vibrational modes of the hydrogel, we were able to predict the refractive indices of the gels from their Raman spectra. This work highlights the importance of a correlative approach through Brillouin–Raman microspectroscopy for the mechano–chemical analysis of biologically relevant samples.

## Introduction

Gelatin hydrogels derived from denatured collagen^1^ constitute a simple model to investigate the physical properties of connective tissue. Gelatin is characterized in a large part by the presence of water, the medium with low compressibility in all biological processes, interspersed within a network of protein molecules, conferring the shear load-bearing property to the system. As such, it is a stable, low-cost, safe, and easy-to-prepare system for optical and biomechanical testing.

Brillouin spectroscopy is a vibrational spectroscopy technique with a unique potential for mechanobiology and biomedical sciences.^2,3^ It is based on the inelastic light scattering effect where incident light is scattered by thermally driven acoustic waves, or “phonons”, which propagate as material density fluctuations resulting in periodic changes in refractive index.^4^ Information on biomechanics is provided both by measurement of the frequency shift, which gives access to the longitudinal elastic modulus, and the linewidth of the Brillouin peak, which yields the attenuation of the acoustic wave and is a measure of the apparent viscosity. Determination of both the longitudinal modulus and apparent viscosity is reliant on knowledge of the refractive index and density of the sample at the same spatial location as the Brillouin measurement. Brillouin microspectroscopy (BM) has proved to be an effective probe of biomechanics (more specifically, micro-viscoelasticity^5,6^) in a range of biological samples, including live cells^7,8^ and organisms,^9,10^ human tissue sections^11,12^ and cornea.^13^ Despite the clear advantages of BM as a nondestructive, contactless probe of micro-biomechanics, it is truly the correlative approach with complementary techniques alongside BM that is most beneficial in enhancing the specificity of the measurements, as it facilitates access to the full information contained within Brillouin spectra. Raman spectroscopy is a promising correlative technique. It provides valuable information on the chemical composition and structure of materials through the inelastic scattering of light from molecular vibrations; hence, it is label-free and chemically specific. We first proposed to interface Brillouin and Raman microspectroscopy^14^ and then realized the first high-contrast Brillouin–Raman microscope,^15^ which enables simultaneous measurement of the micromechanical and chemical properties of samples.^16^ Brillouin and Raman spectroscopy are “sister” techniques, sharing a common optical arrangement and similar light scattering effects, occurring on adjacent frequency scales. In Raman spectroscopy, as well as BM, the signal intensity is linearly proportional to the concentration or density of the scattering species,^17,18^ which in turn is related to the refractive index of the investigated materials.

In the emerging BioBrillouin community, various efforts have been made to assess the refractive indices of samples measured using confocal scanning BM, in order to decouple the optical from the mechanical effects, which contribute to the overall Brillouin line shape. These have been reviewed in recent works,^1,2,19^ so we only recall here that traditional refractometry is insufficient for measuring the refractive index of samples with the micrometric resolution that is required in BM studies. Recent advances in phase imaging have enabled quantitative phase imaging,^9^ holographic phase microscopy^20^ and optical diffraction tomography (ODT)^21,22^ to be implemented alongside BM. Brillouin spectroscopy itself has also been used to determine the refractive index of samples by utilizing two different scattering geometries or angles;^23–25^ however, routine use of these approaches has not yet come to fruition.

In this work, Raman microspectroscopy was successfully applied to gelatin hydrogels, used as biological tissue models, to monitor the refractive index of the gels using vibrational bands. Simultaneous Brillouin and Raman measurements demonstrate how this method can be applied to obtain refractive indices from the same spatial location within the sample, facilitating the determination of the storage and loss moduli from the Brillouin spectra. A calibration model based on Raman band intensities and measurements conducted with an Abbe refractometer^1^ provides access to the refractive index, a finding that can open the full potential of Brillouin imaging in biomedical and life sciences.

## Experimental

Type B gelatin (denatured collagen) was prepared to concentrations between 4 and 18% w/w as previously described.^1,26,27^ The refractive index of all gelatin samples was measured by Abbe refractometry with a D line (589 nm) light source as part of a previous work.^1,26^

The Raman spectra of gelatin at different concentrations were collected using two systems across different frequency ranges. A Renishaw inVia confocal microscope with long working distance 50 × (NA 0.50) objective and using an 830 nm laser was employed for measurements in the “fingerprint” region. Each sample, prepared in a cylindrical mold, was transferred onto a Raman-grade calcium fluoride substrate (Crystran, UK) and analyzed by Raman microspectroscopy. The power at the sample was approximately 130 mW and the backscattered light was dispersed through a 600 lines/mm grating onto a Renishaw deep depletion charge-coupled device (CCD) camera. Raman spectra were acquired with an exposure time of 7 s per spectrum and 32 accumulations. Spectra were analyzed in the range 831–1760 cm^–1^. Three spectra were collected at different locations within the sample for all gel concentrations and WiRE v.4.0 software was used for data acquisition.

A microscope system equipped with a 20 × (NA 0.42) objective and using a 532 nm laser and a Horiba iHR320 Triax Raman spectrometer was used for measurements in the high wavenumber region. The power at the sample was approximately 15 mW. The backscattered light from the sample held in a sealed glass cuvette was split by a short-pass tunable edge filter, which transmitted the quasi-elastic scattered light to a tandem Fabry–Pérot interferometer for Brillouin analysis.^15^ The remaining light was reflected and dispersed through a 600 lines/mm grating onto a CCD camera. Raman spectra of the gels were measured with an exposure time of 1 s and 60 accumulations and Brillouin spectra had an acquisition time of 17 s. Spectra were analyzed in the range 2800–3032 cm^–1^. Five Raman spectra and three Brillouin spectra were collected at different locations within the sample for all gel concentrations. LabSpec5 and JRS GHOST software was used for Raman and Brillouin data acquisition, respectively.

**Figure 1.**
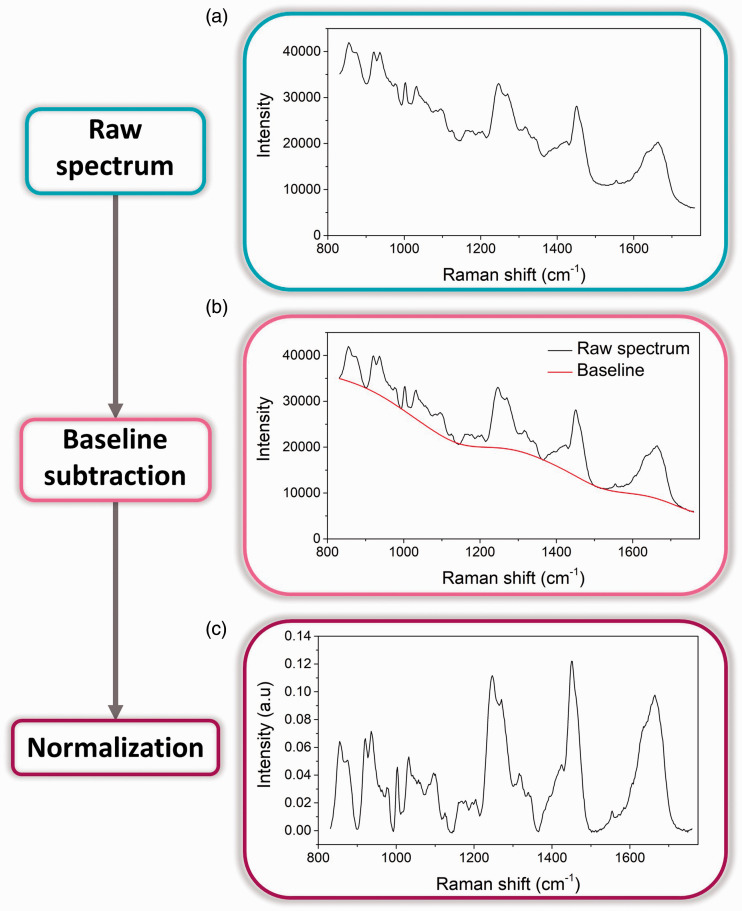
Data processing for an 18% gelatin hydrogel. (a) Raw Raman spectrum. (b) Baseline (red) is determined by asymmetric least squares fit to raw spectrum (black). (c) Spectrum is normalized through division by its Euclidian norm. (No cosmic rays were detected in this measurement).

Raman spectra were processed in Matlab using custom written scripts. Spectral pre-processing (Fig. 1) was performed in three steps: (i) cosmic ray removal, (ii) baseline subtraction using an asymmetric least squares method,^28^ and (iii) normalization of each spectrum to its Euclidian norm.

## Results and Discussion

**Figure 2.**
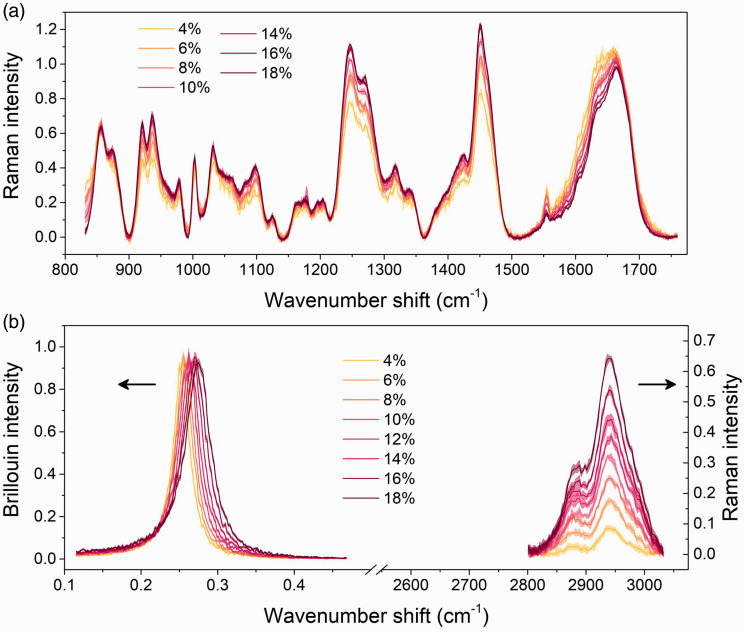
Normalized Raman and Brillouin spectra of gelatins measured across (a) the fingerprint and (b) Brillouin (left) and C–H stretching region (right). Each Raman spectrum is an average from (a) three or (b) five measurements, pre-processed and analyzed as described in the text. Each Brillouin spectrum is an average from three measurements, normalized to the Stokes peak.

Figure 2 shows the evolution in the Raman spectra and simultaneously acquired Brillouin spectra (Fig. 2b) of the gels at varying concentrations across the “fingerprint” and C–H stretching regions.

It can be seen that there is a clear trend in the change of scattering intensity such that, for example, the C–H stretching peaks increase with increasing concentration (Fig. 2b). Brillouin spectra show a blue shift in peak position and an asymmetric broadening as concentration is increased, corresponding to an increase in storage and loss modulus, respectively.^1^

**Figure 3.**
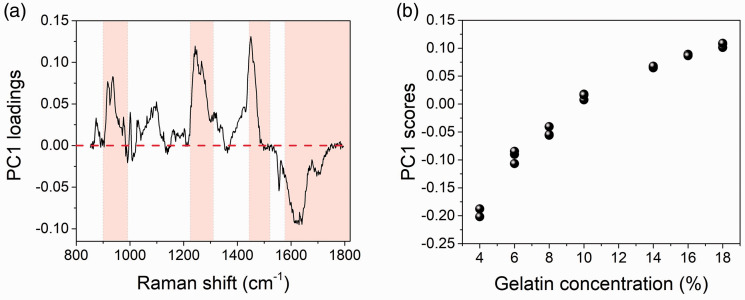
PCA applied to the fingerprint Raman spectra. (a) First principal component (PC1) loading and (b) corresponding score plot. Shading in (a) denotes the spectral regions that express most variance. Tight clustering between repeated measurements at each concentration is observed in (b).

The gelatin spectra in the “fingerprint” region were mean centered and analyzed using principal component analysis (PCA) to determine the spectral regions responsible for the variance of the dataset. The principal components are ranked in such a way that the first component accounts for the highest percentage of the total variance. The first principal component (PC1) accounted for 89% of the total data variance and the loadings (Fig. 3a) highlight the spectral regions where this variation is observed. The corresponding score plot simply represents the trend of the variation described by the loadings versus concentration (Fig. 3b).

The positive loadings (Fig. 3a) result from those peaks that increase in intensity with increasing gel concentration, while the negative loadings are for those signals that decrease in intensity with increasing concentration. The former signals are assigned to the protein component of the gels, while the latter is mainly ascribed to water (see below). It follows that gels of higher water content (4–8% w/w) have negative PC1 scores (Fig. 3b), while those with lower water content ( ≥10%) have positive scores.

Among the spectral regions presenting the largest variance, we can identify the range 898–988 cm^–1^, which contains a doublet at 922 and 938 cm^–1^ (C–C stretching of the proline ring and plausibly C–C stretching of the protein backbone^29,30^) and a small peak at 980 cm^–1^ (arginine^31^). The signals in this region are sensitive to the presence of “bound” water within the hydrogel;^32^ they are indeed found to increase with increasing gel concentration as the number of binding sites increases. An increase in bound water with increasing concentration has already been derived in our previous Brillouin study of gelatin hydrogels.^1^ The range 1216–1300 cm^–1^ presents a doublet at 1248 and 1271 cm^–1^ (amide III^29^), while the range 1431–1507 cm^–1^ corresponds to CH_3_ and CH_2_ deformations.^29,33^ The range between 1562 and 1800 cm^–1^ presents contributions from both protein (amide I^29,33^ centered at 1665 cm^–1^ and assigned to disordered protein structure,^34,35^ with a shoulder at 1635 cm^–1^ associated with denatured triple helices^35^) and water (bending mode at 1635 cm^–1^).^36,37^ Highly hydrated gelatin is expected to be more disordered than gels of lower water content, where a larger proportion of alpha helices are expected to be present.^35^ In addition to the fingerprint region, the C–H stretching band^38^ which presents two peaks at 2885 and 2940 cm^–1^ (symmetric and antisymmetric CH_2_ stretches, respectively) was used in the analysis.

**Figure 4.**
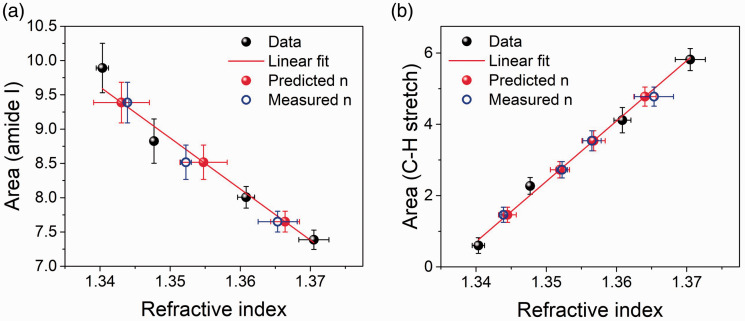
Plot of (a) amide I (1562–1800 cm^–1^) and (b) C–H stretching (2800–3040 cm^–1^) integrated intensity versus refractive index measured with Abbe refractometry. Red line denotes a linear fit of the dataset used as model data for each wavenumber region (black filled circles): (a) R^2 ^= 0.96, (b) R^2 ^= 0.99. Red filled circles denote the refractive indices predicted by the Raman spectra and blue circles indicate those measured using Abbe refractometry for gels of the same concentration. Error bars denote the standard deviation.

Raman signals were integrated with respect to frequency shift, and the intensities obtained were used to build the calibration plots for refractive index analysis. The bands analyzed in this way were the main protein resonances in the fingerprint and high-wavenumber regions, i.e., amide I at 1665 cm^–1^ and the C–H stretching band located between 2800 and 3040 cm^–1^. These band intensities display a linear dependence on gel concentration across the entire range studied here. Similarly, the refractive index of the gels presents a linear dependence on concentration.^1^ This enables a model to be constructed, where the integrated intensities of amide I and C–H stretching bands are plotted versus refractive index (Fig. 4).

In the model, the data are split into two subsets: half of the samples were used as model data to derive the fit and the other half were used as test samples to determine the refractive index. The data points selected as model data corresponded to 4%, 8%, 14%, and 18% gels (black filled circles in Fig. 4). A linear fit was applied to these data (red line) and calibration functions were derived: for the amide I, *y* = 109.3137–74.402 *n*, and for the C–H stretch, *y* = 169.351 *n*–226.226, where *y* represents the integrated intensity of the peak and *n* the corresponding refractive index of the gel. The refractive indices of the test samples, derived from the integrated intensity (red filled circles) using the calibration equations, were then compared with those obtained with an Abbe refractometer^1^ (blue empty circles). Figure 4 shows that there is a close correspondence between predicted and measured values of *n*, confirmed by RMSE (root mean square error) values of 0.002 and 0.0009 for the predicted versus measured refractive indices determined from the amide I and C–H stretching modes, respectively. This indicates that the model is capable of predicting the refractive index of the gelatin hydrogels with high accuracy. Table I lists all results from this analysis.

**Table I.** Refractive indices derived from Abbe refractometry^1^ and Raman measurements using the calibration model.^a^

Gel concentration (%)	Measured ***n*** (±SD)^b^	Predicted ***n*** (±difference)^c^	
		amide I	ν(C–H)
**4**	**1.3403 (±0.0009)**	**1.3363 (±0.004)**	**1.3394 (±0.0009)**
6	1.3403 (±0.0002)	1.3431 (±0.0008)	1.3445 (±0.0006)
**8**	**1.3477 (±0.0006)**	**1.3506 (±0.003)**	**1.3493 (±0.0016)**
10	1.3523 (±0.0008)	1.3548 (±0.003)	1.3519 (±0.0003)
12	1.356 (±0.001)	–	1.3567 (±0.0002)
**14**	**1.361 (±0.001)**	**1.3616 (±0.0008)**	**1.3602 (±0.0007)**
16	1.365 (±0.003)	1.3664 (±0.0011)	1.3641 (±0.0013)
**18**	**1.370 (±0.002)**	**1.3699 (±0.0006)**	**1.3702 (±0.0003)**

a Note that this method is based on a calibration to Abbe refractometry data measured with a D line (589 nm) light source, so all refractive indices presented refer to 589 nm, irrespective of the wavelength at which Raman spectra were acquired.

b Standard deviation derived from five measurements at each concentration.

c Difference between refractive indices measured by Abbe refractometry and those determined from Raman spectroscopy.

d Lines in bold indicate data used for calibration.

A very good estimation of the refractive index is found using this method, with predicted values of *n* being within 0.02–0.3% of the measured values. Differences between measured and predicted values were of the same order of magnitude as the standard deviation of the measurements performed with an Abbe refractometer (Table I). Prediction based on C–H stretching analysis was generally more accurate than that based on amide I, as can be expected because the C–H stretching modes are exclusively protein modes, while the amide I band contains a contribution from the water bending mode (the amide I increases with concentration, while the water bending decreases).

## Conclusion

In summary, we have demonstrated that Raman spectroscopy can be applied to assess the refractive index of biologically relevant samples with appropriate calibration. We have shown that this method can be utilized simultaneously with Brillouin spectroscopy, to assess the localized refractive index from the same spatial location as the Brillouin measurement. The refractive index of gelatin hydrogels displays a linear dependence with concentration, and a similar linear relation is observed for the integrated intensity of the amide I (1562–1800 cm^–1^) and C–H stretching bands (2800–3040 cm^–1^). Using this relation, we have shown that the refractive index can be predicted from Raman spectral intensity to within 0.3% of the value measured with Abbe refractometry, with a higher accuracy observed in the C–H stretching analysis. This is an important result that further substantiates implementations where Raman spectroscopy is applied alongside Brillouin microscopy, as it provides complementary information on the chemical and structural properties of the sample as well as indirectly its refractive index.

There are limitations of this work to note. In fact, the refractive index assessment was performed on a simple model of a biological sample, whereas real specimens such as human tissues are heterogeneous and may present strong discontinuities in refractive index, for example at interfaces. Future investigations into Raman assessments of refractive index in biomedical specimens will be needed to confirm the monitoring capacity of the model. For instance, it remains to be seen how similar approaches can be applied to generate refractive index maps overlaid to Brillouin–Raman images of biological specimens. However, the proof of principle presented here shows great potential for future quantitative Brillouin elastography.
